# Editorial Notes

**Published:** 1907-03

**Authors:** 


### Editorial Notes.

**University College Colston Society.**

The annual meeting of this Society, held on January 11th, under the presidency of Sir William Selby Church, Bart., K.C.B., gave another opportunity for setting forth the claims of the city for the establishment of a Bristol University. The occasion was a notable one, inasmuch as the Lord Mayor of London (Sir William P. Treloar) was entertained as a guest, and, further, it was the first occasion on which the Master of the Society of Merchant Venturers had been present. The Lord Mayor of Bristol and the Sheriff were present, also the Vice-Chancellor of the University of Oxford, the Bishop of Hereford, the Right Hon. Henry Hobhouse, and Sir William R. Anson, Bart., M.P., and many distinguished citizens. The President spoke of Edward Colston's association with St. Bartholomew's Hospital, to which he had been a generous benefactor, and then gave some details of the early history of the Bristol Medical School, and of the distinguished men who had been associated with it, and he trusted that the spirit of Edward Colston might still operate in our midst in furtherance of the establishment of a University for the West of England.

Mr. Henry Hobhouse spoke of the value and need of local Universities, free from the trammels of compulsory Greek, to concentrate their attention on the scientific and modern training, but, he hoped, on the classical side, always ready to seek and obtain aid and inspiration from their older sisters. He spoke of the distinguished career of Sir William Anson, and said it was the fashion in these days to decry University representation. He thought it was a sufficient answer to point to the fact that during the last ten years the Universities had sent to the House of Commons five such men as Lecky, Lubbock, Michael Foster, Jebb, and Anson.

Sir William R. Anson, M.P., dealt with the question of the value of University Colleges under the present educational conditions.

The Vice-Chancellor of the University of Oxford (T. Herbert Warren) admitted that he was a convert to the idea of local and civic Universities. At first he thought it was a pity and a danger to multiply Universities, but he had come to see that that was by no means the case, and that they must bring Universities home to the doors of the people in the large gatherings of population in the great cities. He advocated a University in Bristol for two reasons—because he thought Bristol was the right place for it, and that it would do good to Bristol. They ought to have a University of their own in Bristol, because Bristol was a most admirably-situated place. With its surroundings, the historic associations, the poetry, so to speak, mingling with the prose of commercial prosperity, it was a place filled with opportunities for the education and elevation of the mind, apart from its geographical position. He thought that Bristol might fairly claim to be the next University College to take the rank of a University.

Principal Lloyd Morgan said they looked forward to the time when Bristol would not lightly let distinguished men leave her. They had lost good men in the past, but he hoped they would learn more and more to secure the services of men who came to work with them. At present the College was placed in a difficult position, and he had often been told in America that the position in Bristol was almost impossible. They were preparing students for examinations in which they had no voice, and over which they had no control. The aim and object of education was to bring the individual into vital touch with his environment—into practical touch with his environment. Alluding to the work of the future Bristol University, the speaker pointed out that Bristol was in the midst of agricultural districts. In developing the subject of agriculture, they proposed to have as a beginning one or two professors who were strong both on the scientific and the practical side, and who would be ready to place their knowledge at the disposal of all those interested in the question in the West of England. They would have a strong advisory board, and had been promised the assistance of distinguished experts.

**The University Ideal.**

Professor G. H. Leonard, speaking, February 4th, on "The University Ideal, a chapter from Mediæval History," remarked that, in reference to the prospects of a University for Bristol, one of the greatest things that University would have to do would be to train the imagination of man. Imagination, however, must have something whereon to build, and it was his object to tell, as simply as he could, what a University was, what the Universities of England and Europe had been. The scholar wanted the master, and he found at the University, not only the master, but other students, and the mind was thus sharpened by contact with other minds. In Bristol the University College had always given a cordial welcome to foreigners, the Frenchmen, Germans, and Dutch, and those who hailed from more distant parts and made their home amongst them for the time. He hoped these foreigners would learn something from us, and he wanted them to realise that much was to be learned from them. Many in Bristol believed the University of Bristol, with a colour and character of its own, would attract many foreigners to our city, who would come here, it might be, to study commercial geography, agriculture, literature, or some special school of medicine they would make their own.

**Prof. Sadler on a University for Bristol.**

At the annual distribution of prizes to the students of the Merchant Venturers Technical College, on December 21st, 1906, Professor Michael E. Sadler made a powerful appeal on behalf of a University for Bristol. In the course of his address he remarked that the characteristics of all the new Universities in England were these. Each had one centre for its work. The federal system, by which a University was, so to speak, like a three-legged stool, was, so far as our country was concerned, practically given up. They got more life, more vigour, more municipal pride, more local character if they had a University representing one great city. It was, secondly, characteristic of all those Universities that the examinations should follow the teaching, instead of the teaching being subservient to the examinations, and there was the wise provision by which there was an external and independent examiner. The third characteristic was that they regarded provision for research as absolutely indispensable for the educational vitality of the institution. No teacher could teach properly unless he himself was going on learning. The fourth characteristic was that the Universities were open, without let or hindrance, to men and women of all ranks and all sorts, and, so far as their resources permitted, they would teach anything which the locality thought it was desirable to have taught, and not only in the daytime. One of the great problems we had to face in this country was to enable men who had entered work to take their degree by a systematic series of evening courses. It was a difficult work, because they could not work a competent teacher by day and night ; but it was a problem we had to face and to solve. In this connection he could not but recall to their recollection that in that city, unless he was mistaken, were the first beginnings of that adult school movement, more than one hundred years ago, which, for his own part, he regarded, through its spirit of brotherliness and common service, as perhaps the most remarkable of all the educational movements of our time in this country. But large resources were indispensable to a modern University, and the one thing which made him hesitate in laying this suggestion before them was that he could not disguise from them the fact that year by year the efficiency and maintenance of a great University became more and more expensive. The country would not gain by the establishment of Universities, weak because ill endowed and ill equipped with the right kind of teachers, or with the necessary laboratories and libraries and other buildings which the social life of a great University required. If they took a survey of University life, they realised how elastic things were. They might practically build a University according to the local need, and adjust its organisation to the different requirements of the different districts, but it was highly important, where possible, to unite in one institution the component parts of their University.

Union was good for administrative purposes ; it was good for the interchange of thought between student and teacher ; it was good for the discipline—a much more important part of modern University life than was generally realised; and it was good for future growth, because a single institution appealed more to the imagination, to the State, to the locality, and to the good wishes of the intending benefactor. They wanted one Council, one professional Senate, one academic head. But under these circumstances variety was possible within wide limits. As, for example, at Sheffield, where the Technical College, though an integral part of the University, had its own special traditions and its own history. A great University in Bristol would be the crown of the educational system of the city and district. What we had learnt in the last few years in England had been that national education was one thing from top to bottom with good primary schools, first-rate secondary schools, and great Universities, and, through their reciprocal influence and need, indispensable the one to the other. A University here, well endowed, would furnish Bristol and its neighbourhood with a succession of men fitted to hold the most responsible posts in the scientific enterprise and organisation of modern industry and commerce. And the rapid growth of Bristol encouraged the hope that its citizens would ere long emulate the example of Manchester, Liverpool, Birmingham, Leeds, and Sheffield, and other great cities in building up a modern University. The intellectual influence of a University here would make Bristol, as it used to be in old days, the lantern of the west. The great historical part of Bristol, and of the West Country, would be an inspiring influence in the work of its University. As Burke—perhaps the greatest name connected with Bristol—said, " Our country is an ancient tradition into which we are born." It was our business to blend the old with the new, and a University was one of the centres in which that work could be carried on. And, apart from its intellectual resources, each great city University ought to have halls of residence, in order that the students might enjoy the characteristic benefit of English academic tradition, namely the social training which one got from the give and take on equal terms of collegiate life. And our aim should be to give the rising generation of Englishmen and Englishwomen the best possible opportunity of physical and intellectual training that the world could offer—something which would strengthen and deepen character and fortify will. And we could aim at nothing higher than that the outcome of our education should be fairness of mind combined with capacity for decisive action.

**Sir Michael Foster.**

THE scientific world has sustained a great loss in the death of Sir Michael Foster, K.C.B., F.R.S., D.C.L., M.D. Lond. He possessed a vigorous personality, and as Professor of Physiology at Cambridge for twenty years has exercised a vast influence over the science which has become so completely transformed since the time of Bowman and Carpenter. The scientific development of Physiology in this country has been mainly due to Foster and his pupils, and the University of Cambridge and the Royal Society owe much of their present prestige to the mind and work of the late Member for the University of London. The following personal reminiscences have not been in print elsewhere, and are worthy of record :—

An archæological friend writes : " I well remember a pleasant midsummer day at Cambridge, some years ago, spent in the company of Professor Michael Foster, on the invitation of an intimate friend of his. He met us at the station, and devoted the whole day to showing us the colleges ; he did not seem to care much for the antiquities of the place, and rather treated the subject with subdued scorn. Pointing to the saints in the windows, he said, ' Perhaps they will have me up there one day.'

" At that time he held strong radical views, which he rather impressed on me by saying, with reference to the old saying, ' Whatever is, is right ; ' but I say, ' Whatever is, is wrong.' I was much struck with his genial humour, giving way now and again to peals of laughter.

" After dinner he sent his man to row us on the Cam. He told me that he had been with the Professor for many years, his duty being to anæsthetise the animals for vivisection. He never knew one single case of an animal being allowed to suffer pain, for the animal was always unconscious before the operation began, and was always despatched before it could recover consciousness."

**Cerebro-spinal Fever.**

THE spread of the mysterious and fatal malady known incorrectly as "spotted fever" is causing considerable alarm. Professor Osler states that in New York there have been nearly four thousand cases within the last two years, and that as many as three thousand have been fatal. The necessity for anticipating precautions of comprehensive character is never more evident than in the case of the possible invasion of a district by a comparatively unknown disease. Hence we have endeavoured to give a brief outline of what is known of the disease in an article (page 14) compiled by the Medical Officer of Health for Bristol in conjunction with the Pathologist to the Bristol Royal Infirmary, describing some of the latest known of the salient points in regard to the causation, prophylaxis, and treatment of this disease, and it is hoped that by united action on the part of the profession and of the public authorities, any introduction of cerebro-spinal fever in epidemic form may be promptly averted or checked.

Meantime the name of the disease is of some importance. The term cerebro-spinal meningitis is too comprehensive, inasmuch as it includes all forms of meningitis. The term spotted fever was formerly used as synonymous with typhus, and should still be retained for this disease, in which petechial spots are always present, rather than for a disease in which they are commonly absent. It seems more than likely that many of the earlier epidemics of so-called spotted fever may have been really typhus. Although we rarely see typhus fever now, its name, spotted fever, should not be attached to a malady of which the pathology and bacteriology are totally different.

**Pathogenic Organisms.**

WITHIN the last few months a considerable amount of work has been done upon the metabolic reactions of staphylo-, strepto- and diplococci. The results attained appear to have a practical bearing upon the diagnosis and treatment of infectious conditions, and we hope to present a résumé of the researches and their applications in our next issue.

